# Surveillance of OXA-244-producing *Escherichia coli* and epidemiologic investigation of cases, Denmark, January 2016 to August 2019

**DOI:** 10.2807/1560-7917.ES.2020.25.18.1900742

**Published:** 2020-05-07

**Authors:** Anette M Hammerum, Lone Jannok Porsbo, Frank Hansen, Louise Roer, Hülya Kaya, Anna Henius, Karina Lauenborg Møller, Ulrik S Justesen, Lillian Søes, Bent L Røder, Philip K Thomsen, Mikala Wang, Turid Snekloth Søndergaard, Barbara Juliane Holzknecht, Claus Østergaard, Anne Kjerulf, Brian Kristensen, Henrik Hasman

**Affiliations:** 1 Department of Microbiology and Infection Control, Statens Serum Institut, Copenhagen, Denmark; 2 Infectious Disease Epidemiology & Prevention, Statens Serum Institut, Copenhagen, Denmark; 3 Data Integration and Analysis, Statens Serum Institut, Copenhagen, Denmark; 4 Department of Clinical Microbiology, Odense University Hospital, Odense, Denmark; 5 Department of Clinical Microbiology, Hvidovre University Hospital, Hvidovre, Denmark; 6 Department of Clinical Microbiology, Zealand University Hospital, Slagelse, Denmark; 7 Department of Clinical Microbiology, Aalborg University Hospital, Aalborg, Denmark; 8 Department of Clinical Microbiology, Aarhus University Hospital, Aarhus, Denmark; 9 Department of Clinical Microbiology, Hospital Sønderjylland, Sønderborg, Denmark; 10 Department of Clinical Microbiology, Herlev and Gentofte University Hospital, Herlev, Denmark; 11 Department of Clinical Microbiology, Lillebaelt Hospital, Vejle, Denmark

**Keywords:** OXA-244, carbapenemase, cgMLST, MLST, Escherichia coli

## Abstract

**Background:**

Carbapenemase-producing *Escherichia coli* are increasing worldwide. In recent years, an increase in OXA-244-producing *E. coli* isolates has been seen in the national surveillance of carbapenemase-producing organisms in Denmark.

**Aim:**

Molecular characterisation and epidemiological investigation of OXA-244-producing *E. coli* isolates from January 2016 to August 2019.

**Methods:**

For the epidemiological investigation, data from the Danish National Patient Registry and the Danish register of civil registration were used together with data from phone interviews with patients. Isolates were characterised by analysing whole genome sequences for resistance genes, MLST and core genome MLST (cgMLST).

**Results:**

In total, 24 OXA-244-producing *E. coli* isolates were obtained from 23 patients. Among the 23 patients, 13 reported travelling before detection of the *E. coli* isolates, with seven having visited countries in Northern Africa. Fifteen isolates also carried an extended-spectrum beta-lactamase gene and one had a plasmid-encoded AmpC gene. The most common detected sequence type (ST) was ST38, followed by ST69, ST167, ST10, ST361 and ST3268. Three clonal clusters were detected by cgMLST, but none of these clusters seemed to reflect nosocomial transmission in Denmark.

**Conclusion:**

Import of OXA-244 *E. coli* isolates from travelling abroad seems likely for the majority of cases. Community sources were also possible, as many of the patients had no history of hospitalisation and many of the *E. coli* isolates belonged to STs that are present in the community. It was not possible to point at a single country or a community source as risk factor for acquiring OXA-244-producing *E. coli*.

## Introduction

Carbapenems are used for treatment of infections with multi-resistant Gram-negative bacteria, e.g. extended-spectrum beta-lactamase (ESBL)-producing *Escherichia coli*. Carbapenemase production can be caused by the presence of various carbapenemases. One of the frequently detected carbapenemases in Europe is OXA-48. Besides OXA-48, 17 other variants belong to the OXA-48 carbapenemase group) [1] including OXA-244. In comparison to OXA-48, OXA-244 has a single amino acid substitution (Arg-222-Gly) and has reduced carbapenemase activity [2]. It was first described in Spain from a *Klebsiella pneumoniae* isolate in 2013 [2]. Subsequently, OXA-244-producing *E. coli* isolates were reported from patients in the United Kingdom (UK), France and Egypt, from a healthy person in Germany, from a Dutch traveller and her spouse visiting Indonesia, from river water in Algeria and from an estuary in Lebanon [3-8]. OXA-244-producing *E. coli* can be a challenge for clinical laboratories as they may not grow on selective media used for detection of carbapenemase producers or may not be detected by carbapenemase-specific tests [4]. Furthermore, OXA-244-producing *E. coli* isolates can have low minimal inhibitory concentrations (MIC) for temocillin and meropenem [4,9].

On 18 February 2020, the European Centre for Disease Prevention and Control (ECDC) published a Rapid Risk Assessment (RRA) on the increase in OXA-244-producing *E. coli*, including in Denmark [10]. Before the invitation to contribute data to the RRA, the national reference laboratory at Statens Serum Institute (SSI), Copenhagen had already detected an increase in OXA-244-producing *E. coli* in Denmark, whereas *bla*_OXA-244_ was not detected from other carbapenemase-producing Enterobacterales.

In this study, we characterised by whole genome sequencing (WGS), all OXA-244-producing *E. coli* isolates submitted to SSI during 2016 through July 2019. Data from the Danish National Patient Registry were used for epidemiological investigation together with data from phone interviews with patients infected/colonised with OXA-244-producing *E. coli* isolates from January 2018 through July 2019. Our aim was to investigate if the increase in OXA-244-producing *E. coli* was related to hospital outbreaks or was related to travel from abroad.

## Methods

### Setting

Before 2018, Danish departments of clinical microbiology (DCM) submitted on voluntary basis, carbapenemase-producing organisms (CPO) for verification and genotyping at the national reference laboratory at SSI [11]. On 5 September 2018, the Danish Health Authority made CPO notifiable and published a national guideline to prevent the spread of CPO, including criteria for screening for CPO colonisation after hospitalisation abroad [12]. The CPO isolates are both from screening, e.g. patients travelling abroad or during outbreak investigations, and clinical samples from hospitals and primary care facilities detected during routine susceptibility testing.

Screening isolates are obtained using faecal material from a faecal swabs added to a selective Brain heart infusion (BHI) broth with 0.25 mg/L meropenem. After overnight incubation (18-24 hours) at 35°C, 10 µl from the BHI broth are plated in a selective agar e.g. chromID CARBA SMART (bioMérieux, Ballerup, Denmark) and incubated for 18–24 h at 35°C [13]. Isolates (from clinical and screenings samples) suspected to be carbapenemase-producing according to the European Committee on Antimicrobial Susceptibility Testing (EUCAST) guidelines [14,15] are submitted to SSI from the Danish DCM [11].

### Samples

From January 2016 to August 2019, 24 OXA-244-producing *E. coli* isolates from 23 patients were detected in Denmark as part of the national surveillance of CPO. For patients from whom more than one OXA-244-producing *E. coli* isolate was reported within a rolling 12-month period, only the first isolate was included in the study. From a single patient, two isolates were included as the second isolate carried an additional carbapenemase gene. The numbers of OXA-244 producing *E. coli* isolates were compared with the total numbers of carbapenemase-producing *E. coli* during the same period.

### Epidemiological data and sources

Available data on travel history abroad 6 months before detection of the OXA-244-producing *E. coli* isolates were, as part of the national surveillance, reported by the DCM or by the responsible clinician/physician. Information on age, sex and postal code for residence was collected from the Danish Civil Registration System. Furthermore, patients found positive with OXA-244-producing *E. coli* during January 2018 to August 2019 (or their parents if children under 15 years of age) were interviewed by phone if possible during the summer of 2019, using a questionnaire created for this study containing questions on travelling within 6 months before date of sampling, food consumption and animal contact.

For investigation of possible nosocomial transmission in Denmark, hospitalisation data were retrieved from the DNPR for all patients during the period November 2015 to February 2019. The DNPR data included administration data, diagnoses, examinations and treatment procedures. Administration data included among others; hospital and department codes, dates of admission and discharge on patient level [16]. On 1 February 2019, the coding procedure for DNPR changed. Therefore, DNPR hospital data were only available until 1 February 2019 for epidemiological analysis in the present study.

### Whole genome sequencing and in silico analysis

The genomic DNA was extracted (DNeasy Blood and Tissue Kit, Qiagen, Copenhagen, Denmark), with subsequent library construction (Nextera Kit, Illumina, Little Chesterford, UK) and finally sequenced (MiSeq or Nextseq, Illumina) according to the manufacturer’s instructions to obtain paired-end reads of 2x250 or 2X150 bp in length. Quality control was performed on the raw reads, using the Bifrost pipeline at SSI (https://github.com/ssi-dk/bifrost) with accepted average coverage of 30.00x or above. The WGS data were either used as raw data or de novo assembled using the assemblies generated with SKESA in Bifrost. The raw reads of all 24 isolates were assembled into draft genomes using SKESA version 2.2 in the Bifrost pipeline.

Sequences are available from GenBank under accession number PRJEB36710.

Resistance genes were identified with ResFinder version 2.1 [17] (included in the Bifrost pipeline), using a threshold of 100% identities for identifying genes encoding beta-lactamases and carbapenemases, and 98.00% ID for all other genes encoding transferable antimicrobial resistance. Two different MLST schemes were used, i.e. the Achtman scheme (MLST 1) [18] and the Pasteur scheme (MLST 2) [19].

### Phylogenetic analyses

The contigs of the 24 OXA-244-producing *E. coli* in this study were uploaded to SeqSphere+ version 5.1.0 (Ridom GmbH, Münster, Germany (http://www.ridom.de/seqsphere/)) using the Enterobase *E. coli* cgMLST scheme based on 2,513 genes with a cluster distance threshold of ≤ 10 allele differences.

### Ethical statement

Data on cases were collected as part of the national CPO surveillance according to guidelines for the prevention of spread of CPO by the Danish Health Authority according to which, both patients and treating medical doctor may be contacted for investigation [12]. All data were anonymised and cannot be inferred directly or indirectly to a person.

## Results

### Patients with OXA-244-producing *Escherichia coli*

**Figure 1:**
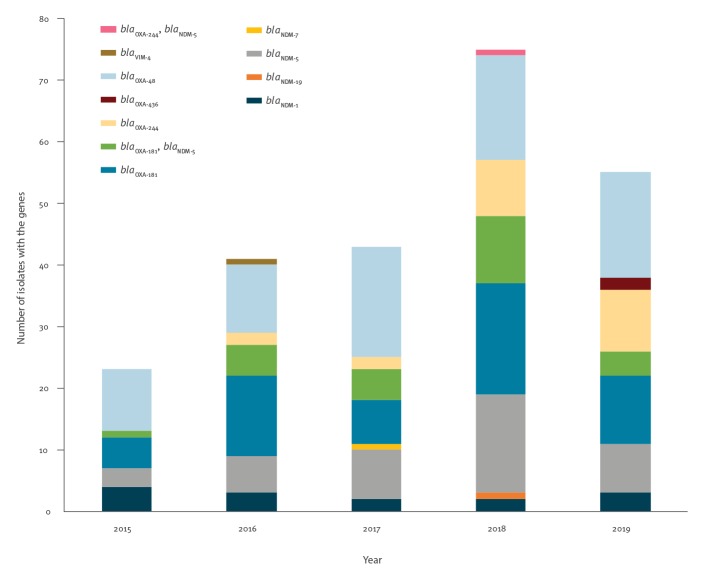
Carbapenemase-producing *Escherichia coli*, Denmark, January 2015–August 2019 (n = 237)

From January 2016 to August 2019, 24 OXA-244-producing *E. coli* isolates were detected in 23 patients in Denmark (Figure 1). From one of the patients, an OXA-244-producing *E. coli* and an OXA-244/NDM-5-producing *E. coli* were obtained from the same faecal sample; both isolates were included in the study (Table 1). The remaining 22 OXA-244-producing *E. coli* isolates were obtained from blood samples (n = 2), urine samples (n = 18) and faecal samples (n = 2).

**Table 1:** Sample information, demographic data and travel history for patients with OXA-244-producing *Escherichia coli*, Denmark, January 2016–August 2019 (n = 23)

Patient number	Sample date	Sample	Sample site	Region	Age group (years)^a^	Sex	Travel^b^
1	May 2016	Urine	General practitioner	Capital	31–50	Female	No travel
2	Nov 2016	Urine	Emergency department	Zealand	51–65	Male	No information
3	Jun 2017	Urine	Hospital	Southern Denmark	31–50	Female	No travel
4	Dec 2017	Urine	General practitioner	Southern Denmark	51–65	Female	No information
5	Feb 2018	Blood	Hospital	Southern Denmark	51–65	Female	Greece
6	Feb 2018	Urine	General practitioner	Zealand	> 65	Female	No
7	May 2018	Faeces	Hospital	Capital	> 65	Female	Egypt
8	Jun 2018	Urine	General practitioner	Capital	3–10	Female	No information
9	Jun 2018	Urine	Hospital	Central Denmark	> 65	Female	No travel
10	Aug 2018	Urine	General practitioner	Southern Denmark	> 65	Female	Tunisia
11	Sep 2018	Blood	Hospital	Zealand	> 65	Male	Poland, Austria
12	Sep 2018	Faeces	Emergency department	Capital	31–50	Female	Egypt, Turkey
13	Oct 2018	Urine	General practitioner	North Denmark	31–50	Female	No travel
14	Feb 2019	Urine	Hospital	Capital	> 65	Female	Egypt
15	Mar 2019	Faeces	Emergency department	Capital	> 65	Male	India
16	Apr 2019	Urine	General practitioner	Central Denmark	> 65	Female	Egypt, Turkey, Italy
17	May 2019	Urine	Hospital	Capital	3–10	Female	Sweden, Portugal
18	May 2019	Urine	General practitioner	Southern Denmark	60	Female	Germany
19	Jun 2019	Urine	General practitioner	Southern Denmark	31–50	Female	Egypt
20	May 2019	Urine	General practitioner	Central Denmark	> 65	Female	No
21	Jun 2019	Urine	General practitioner	Southern Denmark	> 65	Female	Egypt
22	Jun 2019	Urine	Hospital	Central Denmark	51–65	Female	No information
23	Jul 2019	Urine	Hospital	Southern Denmark	> 65	Female	Spain

^a^ The age of the patients ranged from 5 to 79 years, with a median of 58 years.

^b^ Travel 6 months before the OXA-244-producing *Escherichia coli* was detected.

**Figure 2:**
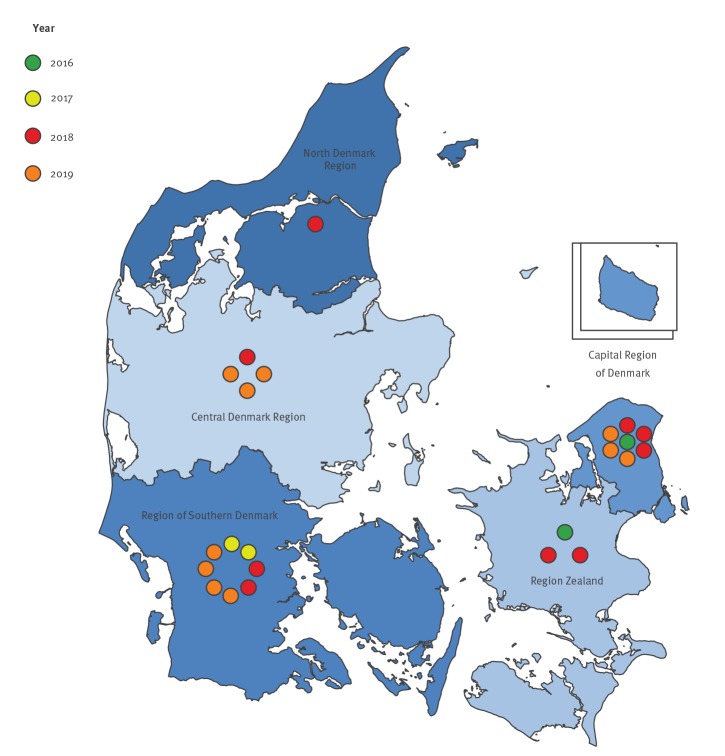
OXA-244-producing *Escherichia coli* by date of detection and region, Denmark, January 2016–August 2019 (n = 24 isolates)

The 24 isolates were collected from hospitals and primary healthcare facilities across all five Danish regions (Figure 2, Table 1).

The age of the patients ranged from 5 to 79 years, with a median of 58 years, 20 were female and three were male. In comparison, during the study period, 63.5% (136/214) of patients with carbapenemase-producing *E. coli* were female in the Danish CPO surveillance, while the majority of the patients with OXA-244-producing *E. coli* in our study were female (p < 0.05).

Of the 23 patients, 13 had been travelling 6 months before detection of the OXA-244-producing *E. coli* isolates, six had no reported history of recent travel and for the remaining four patients, travel information was unavailable (Table 1). Of the 13 patients reporting travel abroad within 6 months before detection of the OXA-244-producing *E. coli* isolate, seven had been to countries in Northern Africa (six to Egypt, one to Tunisia).

During the study period, Carbapenemase-producing *E. coli* isolates were detected from 214 patients; of these, 21 patient samples were taken at a general practitioner, 11 of these patients had OXA-244-producing *E. coli* isolates. Furthermore, OXA-244-producing *E. coli* isolates were detected from the samples from three patients visiting an emergency department and from nine patients hospitalised at different hospital wards.

Data from DNPR were available for the 13 patients with positive samples before February 2019. None of the 13 patients had been hospitalised at the same time at the same hospitals. Among these 13 patients, seven had not been hospitalised in Denmark, from January 2016 to February 2019, and their samples were either from a visit at the general practitioner or at the emergency department.

### Questionnaire of food consumption and animal contact

Fifteen of 19 patients with OXA-244-producing *E. coli* isolates during January 2018 to August 2019 were interviewed on the phone, while four patients were unavailable.

All 15 patients were eating meat and eggs. Four patients had been in contact with cats, 12 with dogs and two with wild birds. None of the patients had been in contact with livestock, hunting animals or reptiles.

### Phylogenetic analysis

The 24 OXA-244-producing *E. coli* isolates belonged to six different STs; ST10 (n = 1), ST38 (n = 13), ST69 (n = 6), ST167 (n = 2), ST361 (n = 1) and ST3268 (n = 1) (Table 2), and 15 had an ESBL gene (*bla*_CTX-M-14b_, *bla*_CTX-M-15_ or *bla*_CTX-M-27_) and one had a plasmid-encoded AmpC gene (*bla*_CMY-2_). Genes encoding colistin resistance, i.e. *mcr* genes, were not detected in any of the isolates. In four isolates, *bla*_OXA-244_ was the only detected resistance gene (Table 2).

**Table 2:** Typing data for OXA-244-producing *Escherichia coli* isolates, Denmark, January 2016–August 2019 (n = 24)^a^

MLST1^b^	MLST2^c^	Patient number	Sample date	Cluster number	Resistance genes (other than *bla*_OXA-244_)						
					Beta-lactam	Aminoglycoside	Fluoroquinolone	Phenicol	Sulphonamide	Tetracycline	Trimethoprim
ST10	ST2	3	Jun 2017	–	*bla*_TEM-1B_	*strA, strB*	–	–	*sul2*	*tet*(A)	*dfrA14*
ST38	ST8	4	Dec 2017	1	*bla*_CTX-M-27_	*aph(3”)-Ib, aph (*6*)-Id, aadA5*	–	–	*sul1,sul2*	*tet*(A)	*dfrA17*
ST38	ST8	5	Feb 2018	1	*bla*_CTX-M-27_	*aph (*6*)-Id, aph(3”)-Ib, aadA5*	–	–	*sul1, sul2*	*tet*(A)	*dfrA17*
ST38	ST8	14	Feb 2019	1	*bla*_CTX-M-27_	*aph (*6*)-Id, aph(3”)-Ib*	–	–	*sul2*	*tet*(A)	–
ST38	ST8	16	Apr 2019	1	*bla*_CTX-M-27_	*aph (*6*)-Id, aph(3”)-Ib*	–	–	*sul2*	*tet*(A)	–
ST38	ST8	17	May 2019	1	–	–	–	–	–	–	–
ST38	ST8	1	May 2016	2	*bla*_CTX-M-14b_, *bla*_TEM-1B_	*aadA1,aph(3')-Ia, strA, strB*	–	–	*sul2*	–	*dfrA1*
ST38	ST8	8	Jun 2018	2	*bla*_CTX-M-14b_, *bla*_TEM-1B_	*aadA1,aph(3')-Ia, strA, strB*	–	–	*sul2*	–	*dfrA1*
ST38	ST8	19	Jun 2019	–	*bla*_CTX-M-27_	*aph (*6*)-Id, aph(3”)-Ib*	–	–	*sul2*	*tet*(A)	–
ST38	ST8	6	Feb 2018	–	*bla*_CTX-M-14b_, *bla*_TEM-1B_	*aph(3”)-Ib,aph (*6*)-Id, addA1*	–	*catA1*	*sul2*	*tet*(D)	*dfrA1*
ST38	ST8	10	Aug 2018	–	*bla*_CMY-2_, *bla*_TEM-1B_	*aph (*6*)-Id, aph(3”)-Ib*	–	–	*sul2*	–	*dfrA8*
ST38	ST8	13	Oct 2018	–	*bla*_CTX-M-14b_, *bla*_TEM-1B_	*aph (*6*)-Id, aph(3')-Ia, aph(3”)-Ib*	–	–	*sul2*	–	*dfrA1*
ST38	ST8	18	May 2019	–	*bla*_CTX-M-14b_, *bla*_TEM-1B_	–	–	*catA1*	–	*tet*(D)	*dfrA1*
ST38	ST8	23	Jul 2019	–	*bla*_CTX-M-14b_, *bla*_TEM-1B_	*aph (*6*)-Id-like, aph(3')-Ia, aph(3”)-Ib-like*	*qnrS1*	–	*sul2*	*tet*(D)	*dfrA14, dfrA1*
ST69	ST3	11	Sep 2018	3	*bla*_TEM-1B_	*aph (*6*)-Id, aph(3”)-Ib*	*QnrS1*	–	*sul2*	*tet*(A)	*dfrA14*
ST69	ST3	21	Jun 2019	3	–	–	–	–	–	–	–
ST69	ST3	22	Jun 2019	3	–	–	–	–	–	–	–
ST69	ST3	9	Jun 2018	–	–	–	*qnrS1*	–	–	*tet*(A)	*dfrA14*
ST69	ST3	15	Mar 2019	–	–	–	–	–	–	–	–
ST69	ST3	2	Nov 2016	–	–	–	*QnrB19*	–	–	*tet*(A), *tet*(U)	–
ST167	ST2	12	Sep 2018	–	*bla*_NDM-5,_ *bla*_CTX-M-15_, *bla*_OXA-1_	*aadA2, aac(6')Ib-cr*	*aac(6')Ib-cr*	*catB4*	*sul1*	–	*dfrA12, dfrA14*
ST167	ST437	12	Sep 2018	–	*bla*_CTX-M-15_, *bla*_TEM-176_, *bla*_OXA-1_	*aph (*6*)-Id, aph(3')-Ia, aph(3”)-Ib, aadA5, aac(6')Ib-cr*	*aac(6')Ib-cr, QnrS1*	*catB4, floR*	*sul2, sul1*	*tet*(A)	*dfrA17, dfrA14*
ST361	ST650	7	May 2018	–	*bla*_CTX-M-14b_, *bla*_TEM-1B_	*aph(3')-Ia,aph(3”)-Ib, aph (*6*)-Id*	–	–	*sul2*	–	*dfrA14*
ST3268	ST535	20	May 2019	–	*bla*_CTX-M-15_, *bla*_TEM-1B_	*aph (*6*)-Id, aph(3”)-Ib*	*qnrS1*	–	*sul2*	*tet*(A)	*dfrA14*

MLST: multilocus sequence typing.

^a^ Samples taken from 23 patients.

^b^ MLST 1: Achtman scheme [18].

^c^ MLST 2: Pasteur scheme [19].

One patient had both an OXA-244-producing ST167 *E. coli* (CPO20180141) and an OXA-244/NDM-5-producing ST167 *E. coli* (CPO20180142), but the two isolates did not cluster together in the cgMLST analysis and they had different resistance gene profiles (Table 2).

Three phylogenetic clusters were detected by cgMLST for the 24 isolates from this study (Table 2).

#### Core-genome multilocus sequence typing clusters

The five isolates in Cluster 1 belonged to ST38 (Table 2). The samples were obtained from December 2017 to May 2019 from three different regions in Denmark. Data from DNPR until February 2019 did not detect any overlapping hospitalisations between the first three patients. Travel information was missing for the first patient in this cluster. Six months prior to detection of the OXA-244- producing *E. coli* isolates, the second patient had been to Greece, the third patient had visited Egypt, the fourth patient had travelled to Egypt, Turkey as well as Italy, and the last patient had been to Sweden and Portugal. There were no geographical associations for place of residence among the patients in Cluster 1.

Both isolates in Cluster 2 belonged to ST38 (Table 2). The isolates were sampled in May 2016 and June 2018 in the same region. The first patient had not been travelling 6 months before detection of the ST38 OXA-244-producing *E. coli* isolate, whereas travel information was missing for the other patient. No epidemiologic links regarding hospitalisation or place of residence were detected between the patients.

The three isolates in Cluster 3 belonged to ST69 (Table 2). The samples were from three different regions and the patients also lived in different parts of the country. Unfortunately, DNPR data were not available for these patients as they all were detected after February 2019. The first patient had been to Poland and Austria 6 months before detection and the second patient had been to Egypt, while travel information was unavailable for the third patient.

## Discussion

In recent years, several hospital-related outbreaks with CPO have been observed in Denmark [11]. The increase in OXA-244-producing *E. coli* in Denmark described here did not appear to be related to Danish hospital outbreaks. Unfortunately, travel information was not available for all patients. Despite this, repeated import from abroad of the OXA-244-producing *E. coli* isolates appears likely. Many of the patients had been travelling 6 months before detection of the OXA-244-producing *E. coli*, predominantly to Northern Africa destinations. Intestinal persistence of CPO for more than 6 months has been reported, so a limitation in our study could by the lack of information about travel prior to 6 months [20]. It could be speculated that the OXA-244-producing *E. coli* could have been acquired during travel abroad longer than 6 months ago (e.g. a single patient reported travel to Egypt 5 years ago, data not shown).

Among the 15 patients interviewed by phone, 12 reported contacts to dogs and four to cats. To our knowledge, OXA-244-producing *E. coli* isolates have neither been reported from dogs nor cats, but a recent study reported on OXA-181-producing *E. coli* isolates obtained from dogs and cats after hospitalisation in a veterinary clinic in Switzerland [21].

All 15 patients interviewed by phone were eating meat, but none of them had been in direct contact with livestock. OXA-244-producing *E. coli* have neither been reported from meat nor from livestock. More generally, CPO are very rarely reported from livestock and meat in Europe. In contrast to Europe, dissemination of CPO among livestock are more common in China, India and Northern Africa [22].

Community sources for the OXA-244-producing *E. coli* isolates seem possible since many of the patients had not been hospitalised and the *E. coli* isolates belonged to STs that are present in the community [23-32]. The ECDC RRA identified one main geographically dispersed ST38 OXA-244-producing *E. coli* cluster with chromosomal-encoded *bla*_OXA-244_ present in all 10 countries, including Denmark, that submitted data for the RRA [10]. The exact location (plasmid or chromosomal) of *bla*_OXA-244_ was not investigated in our study as is would require utilisation of long read sequencing techniques (e.g. MinION and PacBio). The *bla*_OXA-244_ gene can be part of the Tn*51098* transposon [33]. This transposon has been reported at the same location of the chromosomal position among different STs [34].

In conclusion, an increase in OXA-244-producing *E. coli* has been detected in Denmark and other countries in Europe. Import of OXA-244 *E. coli* isolates from travelling abroad seems most likely for the majority of cases. Community sources of infections are also possible, as many of the patients had no history of hospitalisation. Furthermore, many of the *E. coli* isolates belonged to STs that are present in the community. It was not possible to point at a single country or a community source as risk factor for acquiring OXA-244-producing *E. coli* isolates.
